# Retrospective Investigation of the Whole Genome of the Hypovirulent *Listeria monocytogenes* Strain of ST201, CC69, Lineage III, Isolated from a Piglet with Fatal Neurolisteriosis

**DOI:** 10.3390/microorganisms10071442

**Published:** 2022-07-17

**Authors:** Sergey S. Zaitsev, Mariya A. Khizhnyakova, Valentina A. Feodorova

**Affiliations:** Federal Research Center for Virology and Microbiology, Branch in Saratov, 410028 Saratov, Russia; zaytsev-sergey@inbox.ru (S.S.Z.); khizhnyakova_mariya@mail.ru (M.A.K.)

**Keywords:** *Listeria monocytogenes*, neurolisteriosis, whole-genome sequencing (WGS), Oxford Nanopore, MLST, piglet, ST201, CC69, lineage III, antimicrobial resistance, virulence-associated genes

## Abstract

*Listeria monocytogenes* (Lm), the causative agent for both human and animal listeriosis, is considered to be a rare but potentially fatal foodborne pathogen. While Lm strains associated with current cases of human listeriosis are now being intensely investigated, our knowledge of this microorganism which has caused listerial infection in the past is still extremely limited. The objective of this study was a retrospective whole-genome sequence analysis of the Lm collection strain, 4/52-1953, isolated in the middle of the 20th century from a piglet with listerial neuroinfection. The multi-locus sequence typing (MLST) analysis based on seven housekeeping genes (*abcZ*, *bglA*, *cat*, *dapE*, *dat*, *ldh*, and *lhkA*) showed that the Lm strain 4/52-1953 was assigned to the sequence type 201 (ST201), clonal complex 69 (CC69), and phylogenetic lineage III. The strain 4/52-1953, similarly to other ST201 strains, probably originated from the ST9, CC69 via ST157. At least eight different STs, ST69, ST72, ST130, ST136, ST148, ST469, ST769, and ST202, were identified as the descendants of the first generation and a single one, ST2290, was proved to be the descendant of the second generation. Among them there were strains either associated with some sporadic cases of human and animal listerial infection in the course of more than 60 years worldwide or isolated from food samples, fish and dairy products, or migratory birds. Phylogenetic analysis based on whole genomes of all the Lm strains available in the NCBI GenBank (*n* = 256) demonstrated that the strain 4/52-1953 belonged to minor Cluster I, represented by lineage III only, while two other major Clusters, II and III, were formed by lineages I and II. In the genome of the strain 4/52-1953, 41 virulence-associated genes, including the Listeria pathogenicity island 1 (LIPI-1), and LIPI-2 represented by two internalin genes, the *inlA* and *inlB* genes, and five genes related to antibiotic resistance, were found. These findings can help to make the emergence of both hyper- and hypovirulent variants, including those bearing antibiotic resistance genes, more visible and aid the aims of molecular epidemiology as well.

## 1. Introduction

*Listeria monocytogenes*, a facultative, intracellular, potentially fatal foodborne pathogen, the causative agent for both human and animal listeriosis, is prevalent worldwide. This pathogen is widely spread and can be found in different natural, farm and food industry environments, causing infection through contaminated food, meat, seafood, dairy products, water, and livestock feed [1,2,3,4,5]. Listeriosis mainly affects high-risk groups of the human population, such as the elderly, children, pregnant women, newborns, transplant patients, and immunocompromised patients. Infected food-producing animals, including pigs, are an important reservoir for *L. monocytogenes* being the primary source of food contamination followed by foodborne listeriosis outbreaks [5,6,7,8]. Clinically, listeriosis occurs as gastroenteritis which results in: (i) bacteremia, bacterial sepsis, subsequent bacterial infection of the central nervous system, meningitis, and meningoencephalitis, which can lead to lifelong sequelae or death; or (ii) maternal-fetal infection resulting in spontaneous abortion or complications to the pregnancy; and, less often, (iii) endocarditis and spondylodiscitis [9,10,11,12,13,14,15,16,17,18].

According to World Health Organization [19], listeriosis is considered to be a relatively rare disease. However, the high level of mortality, up to 20–30% of patients with Listeria infection, and the detection of *L. monocytogenes* strains with multiple drug resistance, highlight the importance of investigating carefully the causative agent(s) of each Listeria outbreak so as to significantly expand our knowledge of the genetic diversity of the pathogen. This basic data will contribute to the development of novel effective strategies to control the emergence and prevalence of this severe infection worldwide [20].

*L. monocytogenes* is a highly heterogeneous species which has been subdivided via multi-locus sequence typing (MLST) into four evolutionary lineages, almost 30,000 different sequence types (STs) and more than 25,000 clonal complexes (CCs) [21,22,23,24]. The majority of *L. monocytogenes* human clinical isolates belong to lineages I and II. The *L. monocytogenes* strains of III and IV lineages are less often reported, predominantly in ruminants [24,25,26]. The MLST scheme based on the sequences of seven housekeeping genes (*abcZ*, *bglA*, *cat*, *dap*, *dat*, *ldh*, and *lhkA*) has been recognized as the principal method (https://bigsdb.pasteur.fr/listeria/listeria.html, accessed on 7 March 2022), a valuable tool to reconstruct ancestral and evolutionary linkages between *L. monocytogenes* isolates. Further, a routine whole genome sequencing (WGS) can significantly enhance our knowledge of the genetic relationship, origin, spread of individual clonal lines and, overall, aid the phylogenetic analysis of *L. monocytogenes* strains leading to both current and retrospective outbreaks of listeriosis [27,28,29,30].

Nowadays, the prevalence and genetic diversity of *L. monocytogenes* is intensively studied worldwide [31]. However, only a few frequent *L. monocytogenes* clones are considered to be globally distributed [28,32,33]. In fact, our knowledge of other clonal lineages has been extremely limited until now. This is mainly due to the fact that the strains isolated in recent decades are, as a rule, more available compared with the strains isolated earlier. Additionally, the relevant previous cases are not always well-documented. Nevertheless, the retrospective analysis of *L. monocytogenes* strains associated with previous outbreaks of listeriosis can certainly improve present and future food, animal, and public health surveillance, as well as control over the emergence and distribution of original clonal lineage(s) of *L. monocytogenes* and the long molecular evolution of their relevant descendants.

The main goal of this study was to investigate the genome after WGS of the *L. monocytogenes* collection strain isolated in the middle of the XX century from the piglet with listerial neuroinfection. In our research we identified all basic MLST characteristics, the possible ancestor and descendants, antimicrobial resistance genes, and virulence genes for this strain and closely related *L. monocytogenes* isolates of a different origin. We also investigated the phylogenetic relationship of the strain with *L. monocytogenes* of other clonal lineages so as to evaluate the emergence potency for the relevant ST representatives which were detected recently in either human or animal specimens.

## 2. Materials and Methods

*L. monocytogenes* strain 4/52-1953 was obtained from the State Collection of Microorganisms causing dangerous or highly dangerous diseases, including zooanthroponoses, and exotic diseases, of the Federal Research Center for Virology and Microbiology (FRCVM) (https://ficvim.ru/en/, accessed on 29 May 2022). The strain was originally isolated on the 13 January 1953, from a piglet with clinical manifestations of purulent keratoconjunctivitis, eye adhesion, and other symptoms of neuroinfection, such as: apathy, loss of appetite and weight, and fever with increased body temperature up to 40 °C (ranging between 39.5 and 40 °C). The piglet was also uncoordinated and walked in a circle. The animal died on the third day after the beginning of clinical manifestations. The strain was isolated from the brain specimen (postmortem sectional material) by a routine bacterial plate technique as described [34] and designated as *L. monocytogenes* 4/52-1953. The strain was kept frozen at −70 °C. The bacterial culture was plated on Tryptone Soy Yeast Extract Agar (TSYE Agar) (Merck, Darmstadt, Germany) and grown overnight before the experiment.

The total DNA was isolated overnight from the lysate of *L. monocytogenes* strain 4/52-1953 culture as described [34]. The DNA concentration was measured with a spectrophotometer (BioRad Laboratories, Redmond, WA, USA) according to the manufacturer’s instructions. The extracted DNA was sequenced with the Illumina HiSeq 2500 platform (Genoanalytica, Moscow, Russia, https://www.genoanalytica.ru/, accessed on 29 May 2022), related to next generation sequencing (NGS). Preparation of libraries for sequencing on the Illumina platform was carried out using the QIAseq FX DNA Library kit (Qiagen, Hilden, Germany) according to the manufacturer’s protocol. The quality control of the resulting libraries of DNA fragments was fulfilled on a Bioanalyzer Instrument 2100 (Agilent, Santa Clara, CA, USA). Sample preparation on the HiSeq 2500 platform was performed according to the standard manufacturer’s protocols. In parallel, preparation of the DNA library for sequencing was performed using 1D Genomic DNA by ligation SQK-LSK108 (Oxford Nanopore Technologies, Oxford, UK) (MinION) by the standard protocol according to the manufacturer’s recommendations (https://nanoporetech.com/, accessed on 29 May 2022). A FLO-MIN-106 R9.4 Flow cell (Oxford Nanopore Technologies, Oxford, UK) was used to perform sequencing with the MinION and the MinKNOW software.

Hybrid assembly of the *L. monocytogenes* strain 4/52-1953 chromosome by the de novo method was generated with Unicycler v 0.4.9. (https://github.com/rrwick/Unicycler, accessed on 29 May 2022). The complete genome of this strain was deposited at the NCBI GenBank (Acc. number CP048401.1).

Phylogenetic tree based on the comparison of the whole-genome sequences of *L. monocytogenes* strains (*n* = 256) was constructed with the use of the online tool REALPHY 1.13 (https://realphy.unibas.ch/realphy/, accessed on 29 May 2022). Visualization of the phylogenetic tree was carried out using the TreeGraph 2 software [35]. Circular map of the genome visualization was created using the Proksee online tool (https://proksee.ca/, accessed on 29 May 2022).

Multi-locus sequence typing (MLST) of *L. monocytogenes* strain 4/52-1953 based on seven housekeeping genes (*abcZ*, *bglA*, *cat*, *dapE*, *dat*, *ldh*, and *lhkA*) was performed using the BIGSdb-Lm database (https://bigsdb.pasteur.fr/listeria/, accessed on 7 March 2022). The sequence type (ST) was determined by comparison with the relevant allelic profiles for the Listeria isolates available in the BIGSdb-Lm database. The minimum spanning tree construction based on the seven housekeeping genes’ sequences was generated and visualized with the GrapeTree v. 1.5.0 tool 7 (https://github.com/achtman-lab/GrapeTree/releases, accessed on 29 May 2022) as described recently [34,36] in order to demonstrate the phylogenetic relationships between the strain 4/52-1953 and *L. monocytogenes* isolates of different sequence types (STs) available in the BIGSdb-Lm database (https://bigsdb.pasteur.fr/listeria/, accessed on 7 March 2022).

The putative virulence and resistance genes, including metal and disinfectant resistance, antibiotic resistance, stress island, Listeria genomic Islands in *L. monocytogenes* strains 4/52-1953 (Acc. number at the NCBI GenBank: CP023861.1), M7 (Acc. number at the NCBI GenBank: CP002816.1), LM850658 (Acc. number at the NCBI GenBank: CP009242.1), and EGD-e (Acc. number at the NCBI GenBank: CP023861.1) were determined using the BIGSdb-Lm database (https://bigsdb.pasteur.fr/listeria/, accessed on 1 June 2022). Additionally, antibiotic resistance genes were analyzed using The Comprehensive Antibiotic Resistance Database (CARD) (https://card.mcmaster.ca, accessed on 1 June 2022) with Resistance Gene Identifier tools. Next, the prediction of ARM genes in the WGS of *L. monocytogenes* strain 4/52-1953 was performed through the Resistance Gene Identifier (https://card.mcmaster.ca/analyze/rgi, accessed on 29 May 2022). Identification of the resistance genes *fosX*, *lin*, *norB*, *sul,* and *mprF* and determination of their relevant alleles was performed using the Antibiotic Resistance scheme in BIGSdb-Lm database (https://bigsdb.pasteur.fr/listeria/, accessed on 9 June 2022).

## 3. Results and Discussions

In the present investigation, we carefully studied the molecular characteristics of the genome of *L. monocytogenes* strain 4/52-1953, which was first isolated in 1953 from the pathological material of a piglet with clinical symptoms of listerial neuroinfection. For this retrospective study both NGS and MinION platforms were used following the analysis of the relevant genome target genes in comparison with those of *L. monocytogenes* strains available in either the NCBI GenBank (https://www.ncbi.nlm.nih.gov/, accessed on 29 May 2022) or the BIGSdb-Lm database (https://bigsdb.pasteur.fr/listeria/, accessed on 29 May 2022) in order to reconstruct the molecular evolution of the clonal line that caused the death of a farm animal in the middle of the last century.

**Figure 1:**
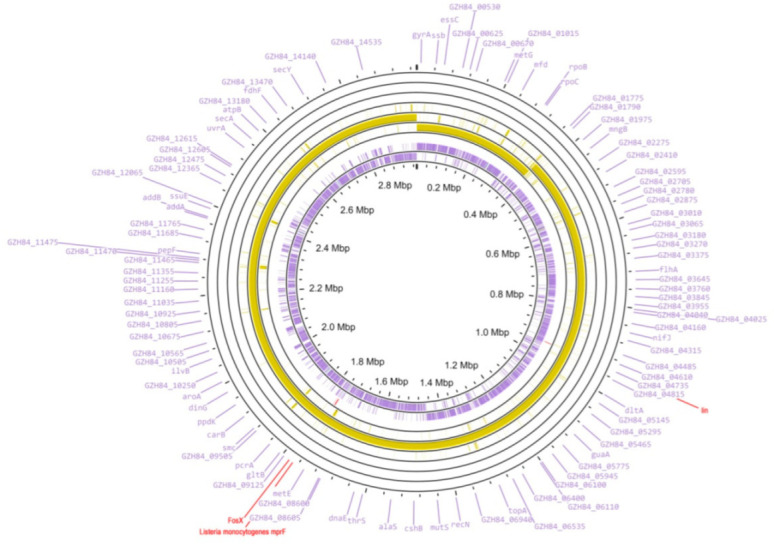
Circular representation of the genome for *L. monocytogenes* strain 4/52-1953. The two circles inside (the purple diagram) show the distribution of the positions for protein-coding genes. The short, red dashed lines in next cycle show the position of ARM genes which were detected using the predictive algorithm CARD RGI (https://card.mcmaster.ca/, accessed on 29 May 2022). The next circles with yellow diagram demonstrate the positions of BLAST hits determined through BLAST comparison of *L. monocytogenes* strain 4/52-1953 with the closest homologous strain *L. monocytogenes* M7. The regions of the genomes of these two strains with 99% homology are marked with a solid yellow line. The regions with low homology (lower than 80%) of the genome of *L. monocytogenes* strain M7 vs. *L. monocytogenes* strain 4/52-1953 are indicated by short, yellow dashed lines. The figure was generated using the Proksee online tool (https://proksee.ca/, accessed on 29 May 2022).

The annotation of *L. monocytogenes* strain 4/52-1953 from the de novo assembled WGS, which was generated with the use of the NCBI Prokaryotic Genome Annotation Pipeline (PGAP), showed the presence of almost 3000 coding sequences (CDSs), and 25 pseudo genes as a result of either frameshift mutations or premature stop codons in the relevant DNA sequences (Figure 1, Table S1).

**Figure 2:**
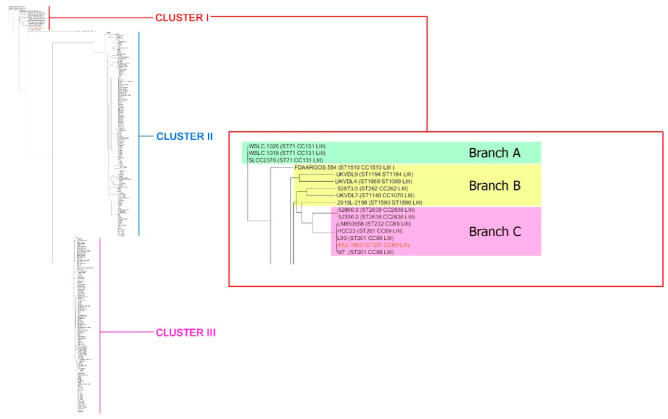
Phylogenetic tree demonstrating the relationship between *L. monocytogenes* strain 4/52-1953 and other 256 whole genomes chromosome sequences of *L. monocytogenes* isolates available in NCBI GenBank (https://www.ncbi.nlm.nih.gov/, accessed on 29 May 2022). *L. monocytogenes* strain 4/52-1953 is highlighted in red. Comparison of full genomes of *L. monocytogenes* demonstrates the separation of strains into three different clades.

In the order to reveal the phylogenetic relationship between *L. monocytogenes* strain 4/52-1953 and other *L. monocytogenes,* we used whole genomes of all the *L. monocytogenes* strains (*n* = 256) available in the NCBI GenBank (https://www.ncbi.nlm.nih.gov/, accessed on 29 May 2022). Phylogenetic analyses of the generated tree demonstrated three distinct Clusters (Figure 2), I, II, and III. In fact, *L. monocytogenes* strain 4/52-1953 belonged to minor Cluster I, represented by lineage III only, while two other major Clusters, II and III, were formed by lineages I and II. Nevertheless, the Listeria representatives of Cluster I can be the possible progenitors of Listeria strains forming Clusters II and III as Cluster I was divided into these two distinct phylogenetic groups.

Cluster I consisted of several branches indicating a certain heterogeneity of Listeria strains. We allocated them, contingently, three branches, A, B, and C (Figure 2, Supplementary Data S1). Branch A was formed by three *L. monocytogenes* strains, two of which, WSLC 1020 and WSLC 1019, were isolated from animal sources, and the third one was reconstructed from the genomic DNA [37]. Interestingly, one of these two strains, WSLC 1020, was found almost 90 years ago (in 1931), which makes it one of the oldest among the Cluster I strains. Branch B was represented by six strains derived predominantly either from biomaterial of ruminants or human clinical isolates with listerial neuroinfection. All these strains were recently (in 2006–2014) revealed in North America. Branch C included seven isolates, the strain 4/52-1953, and six whole genomes of *L. monocytogenes*. Only three of them originated from clinical samples of animals with listerial infection while the other four were detected in either the environmental sample (water) or food (milk, or cheese, or fish). At least four of the seven strains belonging to branch C were close to *L. monocytogenes* strain 4/52-1953 and two others, 52,860 and 52,330, demonstrated the minimal discrimination found in the sub-branch of the same branch (Figure 2). These findings are in agreement with the observations on marked genetic diversity of *L. monocytogenes* strains [20,21,22,23,24,25,27,28,31,32,33,34]. However, in contrast with the early report [24], the isolates belonging to lineage III were detected not only in livestock, but in human cases of listerial infection too [38,39] (Supplementary Data S1).

MLST analysis based on seven housekeeping genes (*abcZ*, *bglA*, *cat*, *dapE*, *dat*, *ldh*, and *lhkA*) showed that *L. monocytogenes* strain 4/52-1953 was assigned to the sequence type 201 (ST 201) of Cluster I. Importantly, all the strains of Cluster I represented a single phylogenetic lineage III. However, only the strains of branch A demonstrated the absolutely identical MLST allele profiles, belonging to a single ST (ST71) and clonal complex (CC131) (Supplementary Data S1). The strains of branch B showed more diversity with a marked polymorphism in the MLST allele profiles, which resulted in marked differences in both STs and CC in six of the six strains. Thus, there were ST262, ST1069, ST1140, ST1194, ST1510, and ST1590 belonging to CC262, CC1069, CC1070, CC1194, CC1510, and CC1590, respectively. Among the strains of branch C only three homologous subgroups, represented by either ST2638 (the relevant clonal complex ST2638) or ST202 (CC69), or ST201 (CC69), were formed. *L. monocytogenes* strain 4/52-1953, similarly with other 20 strains of ST201, differed from the strain of ST202 by the polymorphism only in 1/7 genes, the *ldh* gene. No identical alleles were found between both *L. monocytogenes* strain 4/52-1953 and other strains of ST201 compared with the strains of branches A and B and ST2638 of branch C (Supplementary Data S1). The data obtained are in good correlation with the early reported observations on the possible differentiation of lineage III into several distinct subgroups [24], which in this study were the three separate branches. In fact, according to classification of Moura A. et al. [40], branch A, represented by ST71 only (Supplementary Data S1), strongly corresponded to sublineage 131 (SL131). No correlation was found for branch B, which was formed by six different STs: ST262, ST1069, ST1140, ST1194, ST1510 and ST1590. Similarly, no relevant sublineages were revealed for ST202 and ST2638 of branch C, although sublineage 69 (SL69) was clearly identified for ST201.

The majority (11/21, 52.4%) of ST201 strains were found in Europe in the XXth century, fewer (4/21, 19.1%) in North America, Asia (1/21, 4.8%), and Oceania (1/21, 4.8%). There are no data on the continent/country isolation for 3/21 (14.3%) strains (Supplementary Data S1). Ten of them had an unknown origin (10/21, 47.6%) and the others were isolated from food-producing animals or food samples, fish, and dairy products. For the first time ST201 was detected in 1931 in an unknown part of the world in an animal (https://www.ncbi.nlm.nih.gov/biosample/?term=SAMN02769723, accessed on 29 May 2022). However, subsequently, ST201 strains were isolated almost 20 years later, in the 1950s (1950–1955) in Europe, including Russia. In 1964–1983, ST201 covered North America, Oceania, and Europe and, after a certain period, the strain of ST201 was detected in Asia. Only almost 25 years later, in 2009 (in the XXI century), the next ST201, *L. monocytogenes* strain M7 (Supplementary Data S1), was isolated in another part of the world, China (Zhejiang province), from cow’s milk [41,42]. This may indicate the further evolution of clonal lineage III, ST201, CC69, which includes *L. monocytogenes* strain 4/52-1953. Indeed, in 2016–2017, five *L. monocytogenes* strains of ST201 were detected in migratory black-headed gulls in Dianchi Lake, Kunming, China [43,44]. Importantly, the possible source for all these strains was non-local as they demonstrated a close genetic relationship with the strains from other world regions. Thus, ST201 can be carried and potentially transmitted over long distances by migratory birds, which may present a potential public health risk for both humans and animals [43,44].

The data of MLST analysis based on *L. monocytogenes,* the seven housekeeping genes, and the phylogenetic reconstruction based on *L. monocytogenes* WGSs were closely correlated. In fact, a marked discrimination between *L. monocytogenes* strain 4/52-1953 and the strains with identical phylogenetic and MLST characteristics (ST201, CC69) in comparison with Listeria from other subgroups of Cluster I and the MLST branches A, B, and C, was revealed by both approaches used. These findings supported the conclusion on the extended involvement of the housekeeping genes in the molecular evolution of *L. monocytogenes* [25].

**Figure 3:**
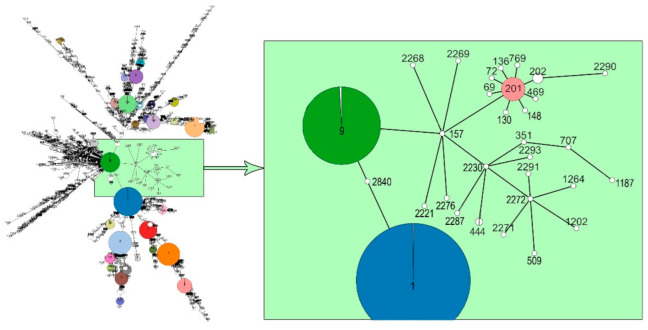
MLST tree minimum spanning tree demonstrating the genetic relatedness between *L. monocytogenes* strain 4/52-1953 (ST201) and all *L. monocytogenes* strains (*n* = 5326) available in the BIGSdb-Pasteur MLST database (https://bigsdb.pasteur.fr/listeria/listeria.html, accessed on 7 March 2022). Each individual circle represents one sequence type (ST). The circle sizes are proportional to the number of strains. Links between circles represent the number of allelic mismatches between individual STs (Supplementary Data S1). The position of ST201 regarding to all STs is shown in green square.

To study the origin and further possible evolution of *L. monocytogenes* strain 4/52-1953 and other strains of ST201, i.e., to identify the potential ancestor(s) and descendants for this clonal lineage, a minimum spanning tree based on the seven housekeeping genes of all *L. monocytogenes* strains available in the BIGSdb-Lm database (https://bigsdb.pasteur.fr/listeria/, accessed on 7 March 2022) was constructed. Figure 3 demonstrates the real genetic relatedness between *L. monocytogenes* strain 4/52-1953 together with other strains of ST201 and other *L. monocytogenes* strains isolated in different periods of XX–XXI centuries worldwide from animal, human, and environmental sources. The most probable main ancestor for *L. monocytogenes* strain 4/52-1953 and ST201 is one of the representatives of the clonal lineage derived from *L. monocytogenes* of ST9, CC9, which was recently assigned to one of the hypovirulent CCs mainly associated with food [45]. According to the main characteristics for ST9 representatives (Supplementary Data S1), all of them clearly belong to CC9, clonal lineage II, and may be isolated from different sources, namely human, animal, food, and either the natural or production environment worldwide. Nevertheless, a minimum spanning tree demonstrated the real genetic relatedness between ST9 of lineage II and *L. monocytogenes* strains of ST201, lineage III, including *L. monocytogenes* strain 4/52-1953, through the only currently detected strain of ST157. However, between *L. monocytogenes* of ST157 and ST201, there must apparently be additional ST(s) which have not yet been found since the comparative analysis of their allelic profiles showed the presence in the nucleotide sequences of each of the seven housekeeping genes from 2 SNPs (in the *dapE* gene) to 70 SNPs (in the *lhkA* gene) (Table S2). Interestingly, the strain of ST157 was isolated in 1939, almost 15 years earlier than *L. monocytogenes* strain 4/52-1953, in North America from an animal source. This makes it possible to suppose that this clonal lineage can really have diverged from ST9 at the beginning; the first third of the XXth century. Probably, ST157 was not distributed widely as only a single strain with this ST has yet been detected. However, to make the final decision more extended, investigations are required. Importantly, ST157 somehow provided the emergence of ST201 and other five clonal lineages of five different STs: ST201, ST2221, ST2230, ST2268, ST2269, and ST2276. Among them only two became the founders for other STs, ST201 and ST2230 (Figure 3). Moreover, ST201 has clearly evolved further by being the group founder for at least eight different STs, ST69, ST72, ST130, ST136, ST148, ST469, ST769, and ST202. In contrast, ST2230 could provide only four novel STs, ST351, ST444, ST2287, and ST2293. Nevertheless, all these strains, the descendants of ST157, were assigned to lineage III only (Supplementary Data S1). These findings can indicate the extended evolution of the strains belonging to lineage III.

Overall, all ST201 descendants of the first generation, ST69, ST72, ST130, ST136, ST148, ST202, ST469, and ST769, available in the BIGSdb-Pasteur MLST database (https://bigsdb.pasteur.fr/listeria/listeria.html, accessed on 7 March 2022) demonstrated identical alleles with six out of seven MLST loci (*abcZ*, *bglA*, *cat*, *dapE*, *dat,* and *lhkA*) and differed from the relevant representatives of ST201 by a single variable allele, *ldh* (Supplementary Data S1). Only ST148, which was isolated in 1924, i.e., earlier than the currently available strains belonging to ST201 and its other descendants, was identical with the group founder, ST201, on the allele *ldh* but differed from it by another single allele, *cat*. Additionally, four strains of ST202, the descendants of ST201, were isolated from animal sources in 1934 at first in the USA and then, in 1967–2009, in New Zealand and Australia (Supplementary Data S1). This may indicate a long and ongoing evolution of the clonal lineage ST201, CC69, which includes *L. monocytogenes* strain 4/52-1953, for no less than 100 years, at least from the 1920s to early 2000s.

All the descendants belonged to identical phylogenetic lineage III and CC69 similarly with the group founder, ST201. However, ST2290, the descendant of ST201 of the second generation through the only currently detected strain of ST202, showed no identical alleles with both ST201 and ST202. Moreover, ST2290 belonged to the novel clonal complex (ST2290) although it was assigned to phylogenetic lineage III similarly with all the descendants of ST201. This enables us to suppose a greater diversity in the spectrum of *L. monocytogenes* allelic profiles and points to ongoing evolution of the clonal lineage with which *L. monocytogenes* strain 4/52-1953 (ST201) is associated.

Surprisingly, the presence of three genes related to antibiotic resistance, *lin* (lincosamides) [46], *L. monocytogenes mprF* (cationic antimicrobial peptides) [47] and *fosX* (fosfomycins) [48] was found in the WGS of *L. monocytogenes* strain 4/52-1953 using the CARD RGI (https://card.mcmaster.ca, accessed on 1 June 2022) tool (Table S3). Additionally, two genes, *norB* (quinolones) [49] and *sul* (sulfonamides) were detected in the genome of this strain (Supplementary Data S2) using the BIGSdb-Lm database (https://bigsdb.pasteur.fr/listeria/, accessed on 1 June 2022). However, no *mprF* was identified by the latter means, although the relevant nucleotide sequence of this gene was identified in the WGS of *L. monocytogenes* strain 4/52-1953 (locus_tag=“GZH84_08960”) with the high homology (98.73%) to the reference gene from the CARD RGI database (https://card.mcmaster.ca, accessed on 1 June 2022). Nevertheless, this observation is very important for understanding the prevalence of antibiotic-resistance genes in *L. monocytogenes* strains isolated from farm animals with neurolisteriosis in the mid-XXth century when the use of antibiotics in both agriculture and public health was not widespread. In fact, it is known that there was an appearance in the same era of microorganisms with marked resistance to antibiotics after their wide use in clinical practice [50]. This may suggest a possible anthropogenic nature of the appearance in animals of Listeria strains carrying antibiotic resistance genes. However, it seems not quite possible because we found only four out of 5312 (0.08%) *L. monocytogenes* strains with identical allele profile for the genes *fosX* (30), *lin* (19), *nor*B (65), and *sul* (23) and no *mprF*, such as: M7, SRR1609997, HCC23, and L99 (Supplementary Data S3). All these strains were isolated either from animal sources (the strains M7, SRR1609997 and HCC23) or food (L99), and belonged to phylogenetic lineage III and CC69. No similar allele profile for the genes *fosX*, *lin*, *norB*, *sul,* and *mprF* have been available in the BIGSdb-Lm database (https://bigsdb.pasteur.fr/listeria/, accessed on 9 June 2022) for *L. monocytogenes* strains derived from a human source (Supplementary Data S3). On the other hand, the presence these genes with identical allele profiles in *L. monocytogenes* strain SRR1609997 which was isolated from an animal source in 1931, i.e., before the ‘antibiotic era’ can support of the idea of an anthropogenic factor in the evident transfer from human to animals of, at least, these five antibiotic-related genes. However, to make a final decision there must be more information on the distribution of resistance genes in *L. monocytogenes* strains circulated in the first part of the XX century in both animals and humans worldwide.

Importantly, at least 41 virulence-associated genes indicated in the virulence gene scheme by the Institute Pasteur [40] were identified in the genome of *L. monocytogenes* strain 4/52-1953 (Supplementary Data S2). Listeria pathogenicity island 1 (LIPI-1), and LIPI-2 represented by two internalin genes, the *inlA* and *inlB* genes, encode proteins involved in the invasion of host cell by pathogen [51,52]. No LIPI-3 (*llsAXGHBYDP*) and LIPI-4 (*licABC*, *lm900558-70013* and *glvA*) were identified, although all these genes, except *comK* (LMOf2365_2303), were easily detected in the genome of *L. monocytogenes* strain EGD-e which was used as the reference one [40]. In contrast to the reference strain, no Listeria genomic islands, stress island, and metal and detergent resistance genes were found in the strain 4/52-1953, so this microorganism could not be classified as hypervirulent *L. monocytogenes* strain [53,54]. Interestingly, *L. monocytogenes* strain M7 belonging to the same ST201, and isolated almost 60 years later than 4/52-1953 demonstrated the identical spectrum of the virulence-related genes (Supplementary Data S2). This may indicate no marked evolution for these representatives of ST201. However, in contrast to the nonpathogenic strain M7 [42], the strain 4/52-1953 was definitely associated with the outbreak of animal neurolisteriosis. Notably, the strain LM850658 of ST202, the descendant of the ST201, also demonstrated an identical spectrum of virulence-associated genes with the exception of only the *agrA* gene (*lmo0051*), directly involved in *L. monocytogenes* virulence because of the deletion mutants (*ΔagrA)* which were attenuated on mouse model [55]. This may suggest a weak tendency to partial restoration of virulent activity for some descendant(s) of ST201. However, to resolve this hypothesis, more descendants of ST201 need to be carefully investigated. The great limitation of our study is the availability only of a single genome of these descendants, the strain (ST202), for our investigation. No other WGSs for ST201 descendants including ST are now in the GenBank database (https://www.ncbi.nlm.nih.gov/, accessed on 29 May 2022) to provide the relevant extended research. Nevertheless, the extended study of a larger number of *L. monocytogenes* strains of ST201 and the relevant descendants is rational and very valuable in the future for the aims of molecular epidemiology.

## 4. Conclusions

The retrospective analysis of the whole-genome sequence of *L. monocytogenes* strain 4/52-1953 helped to greatly improve our knowledge of the molecular evolution and genetic diversity of *L. monocytogenes* strains belonging to the relevant clonal lineage. This strain, the causative agent for the fatal animal neurolisteriosis, belonged to the phylogenetic lineage III, ST201, CC69. In the current study we carefully investigated the possible descendants of the relevant ST201. In fact, among them there were strains either associated with some sporadic cases of human and animal listerial infection over more than 60 years worldwide or isolated from food samples, fish, and dairy products or migratory birds. The presence of only LIPI-1 and genes for two internalins, the *inlA* and *inlB*, was identified in the genomes of the strain 4/52-1953 and other representatives of this lineage which should be classified as hypovirulent ones. However, at least five antibiotic resistance genes were found in all the genomes tested. These findings may indicate the importance of a careful investigation of individual genomes of *L. monocytogenes* isolated both currently and under previous cases of listeriosis to cover the gapes in our knowledge on Listeria evolution and molecular epidemiology. Thus, our future research will be devoted to investigating an extended number of *L. monocytogenes* strains of ST201 and the relevant descendants to unravel the emergence of both hyper- and hypovirulent variants, including those bearing antibiotic resistance genes.

## Data Availability

Data presented in this study are available upon request.

## Supplementary Materials

The following supporting information can be downloaded at: https://www.mdpi.com/article/10.3390/microorganisms10071442/s1, Table S1: Brief characteristics of *L. monocytogenes* strain 4/52-1953 after the automatic contig annotation that was generated based on the NCBI Prokaryotic Genome Annotation Pipeline (PGAP). Table S2: The number of SNPs in *L. monocytogenes* housekeeping genes of ST 201 versus ST157. Table S3: Antibiotic resistance genes identified using the CARD RGI tool in the whole-genome sequence of *L. monocytogenes* strain 4/52-1953. Supplementary Data S1: Brief phylogeny of *L. monocytogenes* strain 4/52-1953 and other ST201 strains. Supplementary Data S2: List of the main virulence-associated genes identified in the genome of *L. monocytogenes* strain 4/52-1953 using the virulence gene scheme by the Institute Pasteur. Supplementary Data S3: Detection of the resistance genes *fosX*, *lin*, *nor*B, *sul* and *mprF* in *L. monocytogenes* strains. References [56,57] are cited in Supplementary Materials.
