# Supplementary material for: Retrospective Investigation of the Whole Genome of the Hypovirulent Listeria monocytogenes Strain of ST201, CC69, Lineage III, Isolated from a Piglet with Fatal Neurolisteriosis
Source: Microorganisms. 2022 Jul 17;10(7):1442. doi: 10.3390/microorganisms10071442 (PMC9324732; doi:10.3390/microorganisms10071442)
Supplement: Supplementary file 1 [file microorganisms-10-01442-s001.zip › Feodorova_Supplementary.pdf]

Table S1. Brief characteristics of the *L. monocytogenes* strain 4/52-1953 after the automatic contig annotation that was generated based on the NCBI Prokaryotic Genome Annotation Pipeline (PGAP).

Number of Genes						Number of Pseudo Genes						GC, %
**Totally**	**CDSs**	**Coding proteins**	**tRNAs/complete tRNAs**	**ncRNA**	**CDSs (without proteins)**	**Total**	**Ambiguous residues**	**With frameshift**	**Incomplete**	**With internal stop**	**Multiple problems**	
3.027	2.950	2.925	5, 5, 5 (5S, 16S, 23S)	4	25	25	0 of 25	11 of 25	14 of 25	3 of 25	3 of 25	38.17

Table S2. The number of SNPs in the *L. monocytogenes* housekeeping genes of ST 201 versus ST157.

abcZ	*bglA*	*cat*	*dapE*	*dat*	*ldh*	*lhkA*
10	7	5	2	3	3	70

[1] BIGSdb-Lm provides access to genotypic data for *L. monocytogenes* isolates based on multi-locus sequence typing (MLST) (https://bigsdb.pasteur.fr/listeria/, accessed on 01 June 2022).

Table S3. Antibiotic resistance genes identified using the CARD RGI tool in the whole genome sequence of *L. monocytogenes* strain 4/52-1953.

RGI Criteria	ARO Term	Detection Criteria	AMR Gene Family	Drug Class	Resistance Mechanism	% Identity of Matching Region	% Length of Reference Sequence
Strict	*lin*	protein homolog model	lincosamide nucleotidyltransferase (LNU)	lincosamides	antibiotic inactivation	98.28	100.00
Strict	*L. monocytogenes mprF*	protein homolog model	defensin resistant mprF	peptide antibiotics	antibiotic target alteration	98.73	100.00
Strict	*fosX*	protein homolog model	fosfomycin thiol transferase	fosfomycins	antibiotic inactivation	93.98	100.00
